# Perspective: Did Covid-19 Change Non-small Cell Lung Cancer Surgery Approach?

**DOI:** 10.3389/fsurg.2021.662592

**Published:** 2021-05-12

**Authors:** Paola Ciriaco, Angelo Carretta, Alessandro Bandiera, Piergiorgio Muriana, Giampiero Negri

**Affiliations:** ^1^Department of Thoracic Surgery, Vita-Salute San Raffaele University, Milan, Italy; ^2^IRCCS San Raffaele Scientific Institute, Vita-Salute San Raffaele University, Milan, Italy

**Keywords:** non-small cell lung cancer, surgery, Covid-19, pandemic, emergency

## Abstract

The novel coronavirus (Covid-19), as of January 2021, infected more than 85 million people worldwide, causing the death of about 1,840 million. Italy had more than 2 million infected and about 75,000 deaths. Many hospitals reduced their ordinary activity by up to 80%, to leave healthcare staff, wards, and intensive care unit (ICU) beds available for the significant number of Covid-19 patients. All this resulted in a prolonged wait for hospitalization of all other patients, including those with non-small cell lung cancer (NSCLC) eligible for surgery. The majority of thoracic surgery departments changed the clinical-therapeutic path of patients, re-adapting procedures based on the needs dictated by the pandemic while not delaying the necessary treatment. The establishment of Covid-19-free hub centers allowed some elective surgery in NSCLC patients but most of the operations were delayed. The technology has partly facilitated patients' visits through telemedicine when security protocols have prevented face-to-face assessments. Multidisciplinary consultations had to deal also with the priority of the NSCLC cases discussed. Interpretation of radiologic exams had to take into account the differential diagnosis with Covid-19 infection. All the knowledge and experience of the past months reveal that the Covid-19 pandemic has not substantially changed the indications and type of surgical treatment in NSCLC. However, the diagnostic process has become more complex, requiring rigorous planning, thus changing the approach with the patients.

## Introduction

The novel coronavirus (Covid-19) since its spread in late 2019 has caused a high number of deaths and infections worldwide. All hospital strategies aimed at containing the infection by introducing isolation protocols (1–3). Many elective procedures and operations were postponed despite other diseases such as non-small cell lung cancer (NSCLC) continued to be an important cause of death (4–9).

The factors that have led to a major change in the management of patients with lung cancer are manifold. The reduction in the availability of hospital beds has led to a delay in medical and surgical care for patients with lung cancer (10). Screening programs were temporarily interrupted and patients did not feel confident about going through regular visits and follow-ups (4, 6, 11, 12).

Several centers have reorganized their activity to cope with the surgical treatment of NSCLC in an emergency due to the Covid-19 pandemic (5, 13). Diagnostic-therapeutic paths have been improved, allowing an adequate and timely treatment of the NSCLC.

## Diagnosis of Covid-19

The preferred current diagnostic method for Covid-19 according to the WHO and Centers for Disease Control and Prevention (CDC) is the detection of SARS-CoV-2 nucleic acid in patients specimen (molecular test RT-PCR) (5). SARS-Cov-2 proliferates generally in type II alveolar cells and the viral peak appears 3-5 days after the onset of disease. Samples are obtained from the upper airways (nose, pharynx) and the lower airway (sputum, bronchoalveolar lavage). The rapid version is also available (14).

Rapid antigenic tests are currently used too to look for the presence of viral proteins able to bind to antibodies. Positivity or not is like a kind of on-off signal. Rapid antigenic tests can be performed on a sample taken by swab or through saliva; the result is almost immediate and does not require healthcare personnel and laboratory tools. In addition to being low in cost, the test could be used in situations, such as schools or airports, where many subjects need to be tested quickly. As for the rapid molecular tests the limit is represented by the reliability, which still needs to be improved (14).

Covid-19 infection should be also considered when clinical symptoms and radiologic patterns are suggestive even in absence of positive testing (15). Several European hospitals suggest a diagnostic algorithm in which chest x-ray is described as a first-line triage tool. Chest CT scan following chest x-ray demonstrates typical imaging features in patients with Covid-19, including bilateral ground glass opacities (15).

## Pre-Operative Screening

Currently, the most used screening method is the RT-PCR nasopharyngeal swab. In some centers it is followed (4, 11, 16) or preceded (17) by a chest CT scan to look for images suggestive of Covid-19 infection.

According to a survey carried out by Depypere in April 2020 among the European Society of Thoracic Surgeons (ESTS) members, 23% of the centers interviewed performed the search for SARS-Covid-2 infection only in patients who developed in-hospital symptoms. At the time of awareness of the spread of the virus, the screening of surgical patients was carried out on all patients in 25% of the centers interviewed and only in hospitalized one's in 23% of the centers (11). Sporadic centers opted for RT-PCR nasopharyngeal swabs to be performed in all patients at the time of discharge (12).

Positive anamnesis for Covid-19 symptoms despite a negative nasopharyngeal swab is considered in many centers suspicious for viral infections and thus, further investigated using chest x-ray and/or CT scan.

## Experience at the Department of Thoracic Surgery of the University Hospital San Raffaele, Milan

At the beginning of the pandemic, in our department, RT-PCR nasopharyngeal swabs were offered only to symptomatic hospitalized patients and to those with a history of close contact with patients resulted positive for Covid-19. Molecular tests were performed on healthcare personnel only in case of suspected contact with Covid-19 positive patients.

From March 2020, all patients undergo RT-PCR nasopharyngeal swab 48-72 h before hospital admission, while healthcare personnel receives a rapid antigenic test every 2 weeks.

Patients with a suspicious chest CT scan, despite a negative nasopharyngeal swab, were treated as infected and therefore isolated.

## Surgery Planning

The spread of the pandemic has forced hospitals to reorganize their activities. Although a low percentage of patients needed a place in the intensive care unit (ICU), the widespread of the infection led to a rapid saturation of available beds and forced many departments to transform into Covid-19 care wards (18, 19). Thoracic surgery for NSCLC has been considerably reduced in 48% of the European centers because of minimal ICU beds available and shortage of personnel that in 63% of the centers was involved in the cure of Covid-19 patients (11).

All thoracic surgeons faced the necessity to create a case triage system for cases of NSCLC without risking the disease progression due to the delayed surgery. The majority of the hospitals created separate pathways for Covid-19 patients, dedicated operating rooms, and provided the healthcare personnel with the most appropriate personal protective equipment (20).

The limited availability of places, however, certainly did not allow the rapid disposal of the operating lists of the major thoracic surgery centers (2, 21, 22).

According to a survey carried out among the ESTS members by Depypere, surgical planning was considered affected by the pandemic “extremely and/or considerably” in 55.5% of the centers (11). The planning was mostly affected by the availability of beds in the ward, reduced staff, and by the results of nasopharyngeal swabs. The reduced availability of ICU beds was irrelevant for 35.8% of the centers. Most likely, half of the centers that were not particularly affected in their surgical planning were those in which the first wave of the pandemic was not significant.

Indication for surgical treatment of NSCLC remained the same throughout the pandemic; however, for the above reasons elective surgery has been somewhat delayed (5). The American College of Surgeons has published Covid-19 elective case triage guidelines for surgical cases based on hospital resources available depending on the phase of the pandemic (23). In a semi-urgent setting, they recommend that surgery should be given to patients whose survivorship is likely to be compromised if the operation is not performed within 3 months.

Specifically for lung cancer, such cases include solid or predominantly solid (>50%) nodules, presumed lung cancer > 2 cm, or N1 positive. Patients who finished induction therapy are included in the category too. In an urgent setting, surgery is restricted to patients likely to have survival compromised if the operation is not performed within the next few days (23).

In detail in Lombardy, Italy (24), lung cancer patients were categorized into two groups. Red code (group I) patients with stage IC, II, III or patients who already received induction therapy. In this group, surgery should be guaranteed in 4 weeks. Yellow code (group II) patients in stage I with nodules <2 cm or indolent malignancies that could be postponed for 1 or 2 months (24). The Lombardy region selected several theoretically Covid-free hospital hubs to divert red code patients, but the reduced number of spaces compared to the conspicuous demand, however, caused delays in surgical treatment.

To comply with regional regulations, multidisciplinary decisions, have been influenced by the degree of priority to assign to NSCLC cases, without changing indication and treatment. Approximately 54.5% of the centers interviewed by Depypere reported to have been influenced by the degree of priority, while 11.7% of centers reported that they were not at least affected by the current situation (11).

The pandemic has also changed contacts with patients and colleagues. At least in the initial phase, screening programs were interrupted and face-to-face visits were reduced to a minimum. The technology has partly facilitated patients' visits through telemedicine when security protocols have prevented face-to-face assessments. Technology has partly filled this gap with the possibility of using telemedicine (25). More than 50% of the centers involved in the Depypere survey, reported using telemedicine for both pre-operative and post-operative contact with patients (11). However, in patients who need closer supervision, such as pre-operative patients, this procedure might have some limitation.

Technology improved the scientific updating since it was facilitated by the numerous virtual meetings which, not requiring physical travel, can be followed by a greater number of specialists.

In a Covid-19 positive patient, surgery should be delayed for 2-3 weeks (5, 26) unless it is an emergency. In this case, the health care personnel must carry out the intervention in a dedicated Covid-room and with the necessary precautions.

Depypere reports that more than 59% of the centers postponed lung resection for 2 weeks in the event of a positive Covid-19 finding. The survey, reports also that the total number of Covid-19 patients undergoing lung resection for cancer at the time of April 2020, ranged from 30.6 to 22.7% of regular cases (11).

Scrolling through the literature, some patients undergoing lung resection for cancer were occasionally discovered to be Covid-19 positive (12, 27, 28). Smelt et al. (27) and Mejía et al. (12) report in their small series that occasional post-operative finding of Covid-19 positive swab in these patients did not appear to adversely affect their course. Bilkhu and Billè, on the other hand, states, “Post-operative mortality after thoracic surgery in patients with Covid-19 is significantly higher than the standard reported mortality” (4). It is important to point out that occasional perioperative finding of a RT-PCR positive swab belongs mainly to the beginning of pandemic when, in many centers, adequate safety protocols were still lacking (29, 30).

The improvement of the pre-operative screening in the second part of the pandemic has made it possible to drastically reduce the number of occasional findings of Covid-19 positive in NSLC hospitalized patients.

## Experience at the Department of Thoracic Surgery of the University Hospital San Raffaele, Milan

Our hospital, a large tertiary one and research center, was immediately involved in the management of the public health emergency. Since the beginning, the elective surgical activity of the hospital, including thoracic surgery, was reduced and most of the departments have been reorganized as Covid-19 areas. At the same time, the hospital became the regional referral hub for cardiovascular emergencies. The number of ICU beds was increased from 28 to 72 of which 18 dedicated to cardiovascular emergencies. All the involved health care personnel was trained to use personal protective equipment and to manage Covid-19 patients in both ward and ICU (13). This scenario lasted until June 2020 when the normal activities were resumed. With the appearance of the second wave of Covid-19 in October 2020, the same procedure of reshaping was rapidly retraced although with a lower load of Covid-19 patients.

Our thoracic surgery department suffered a 50% reduction in beds at the two peaks of the pandemic. However, the number of surgeries compared to the same periods of the previous year has not undergone substantial changes (Table 1).

**Table 1:** Surgical activity during the two phases of Covid-19 pandemic compared to the previous year.

	**2019**	**2020**
**February-May, total**	**152**	135 (124 + 11^*^)
Major pulmonary surgery, total	*38*	40 (35 + 5^*^)
Minor surgery, total	*61*	64 (58 + 6^*^)
Pulmonary	25	23 (21 + 2^*^)
Others	36	41 (37 + 4^*^)
Endoscopic procedures, total	*53*	*31*
EBUS-TBNA	18	12
ENB ± fiducial markers	6	3
Tracheal/bronchial dilatation, rigid bronchoscopy	12	7
T-tube/tracheostomy cannula change	11	6
Others	6	3
**September-December, total**	**140**	138 (137 + 1^*^)
Major pulmonary surgery, total	*34*	40 (39 + 1^*^)
Minor surgery, total	*62*	*63*
Pulmonary	26	29
Others	36	34
Endoscopic procedures, total	*44*	*35*
EBUS-TBNA	14	10
ENB ± fiducial markers	8	1
Tracheal/bronchial dilatation, rigid bronchoscopy	9	14
T-tube/tracheostomy cannula change	10	6
Others	3	4

* *Patients undergoing surgery for non-small cell lung cancer at the Covid-free hub. Bold values indicates total of surgical interventions. Italic values indicates number of interventions for each specific procedure*.

In our experience, we diverted NSCLC patients requiring major resection at the Covid-free hub, in 4.3% of cases (12 patients/273), recording a minimal impact on the entire surgical activity for NSCLC in the Covid-19 era (Table 1).

**Figure 1:**
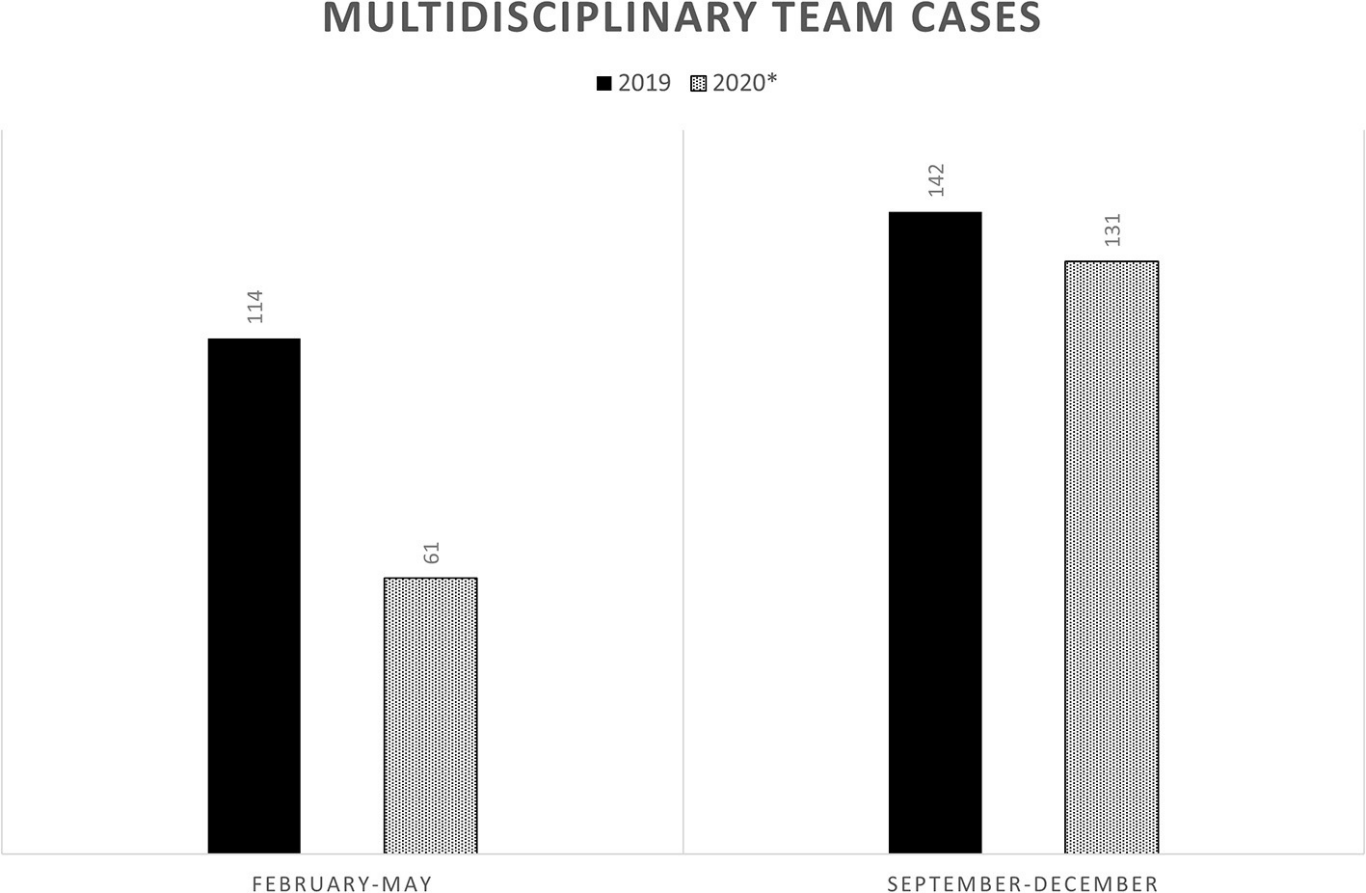
Multidisciplinary team cases of non-small cell lung cancer discussed in the two phases of Covid-19 pandemic and compared with the previous year.

Multidisciplinary decisions, in our experience, have been influenced by the degree of priority to assign to NSCLC cases, without changing indication and treatment. The meetings were carried out virtually. The was a decrease in discussed cases only in the first phase of the pandemic, returning to normal compared to the previous year, in the second wave (Figure 1).

In the first phase of the pandemic, in our department, there were cases of Covid-19 positive patients undergoing surgery. Only one presented with NSLC. A 75 years old patient underwent lung resection for cancer and was post-operatively discovered Covid-19 following a suggestive chest x-ray. The infection was confirmed by RT-PCR nasopharyngeal swab. The patient was transferred to a Covid-dedicated ward and discharged after 25 days, he remained asymptomatic throughout hospitalization. He is still alive and disease-free at 10 months follow-up.

## Conclusions

The Covid-19 pandemic did not change surgical indication in NSCLC but it has definitively changed the way of approaching patients.

There is an increase in expanding telemedicine capabilities for long-term follow-up and first consultation.

Multidisciplinary teams are tasked with verifying not only the indication and cure of NSCLC but also surgical treatment priorities.

Cancer centers should be organized for triage and screening tests using a RT-PCR nasopharyngeal swab. In patients presenting Covid-19 suggestive symptoms but negative swab, radiological and serological investigations should be deepened.

Hospitals must be reorganized to ensure the division between Covid and non-Covid patients and the healthcare personnel should be equipped with adequate personal protective devices.

The possibility to join virtual meetings gives to all centers the possibility to update for all the news concerning this topic and beyond and should be maintained even after the pandemic will be resolved.

The hope shared by the entire scientific community is that both Covid-19 effective care and the vaccine can lead us back to a pre-pandemic surgical activity while treasuring the innovative technological aspects in communication among colleagues and with patients.

Anyhow, all hospitals will benefit even later from the organization that resulted from the Covid-19 pandemic.

## Data Availability Statement

The raw data supporting the conclusions of this article will be made available by the authors, without undue reservation.

## Author Contributions

PC was responsible for the type of paper, data collection, drafting, and writing of the paper itself. AC was responsible for data collection and revision of the paper. AB was responsible for literature review and data collection. PM was responsible for literature review and revision of the paper. GN as chief of the department of thoracic surgery of the San Raffaele Hospital coordinated the activities and reviewed the paper. All authors contributed to the article and approved the submitted version.

## Conflict of Interest

The authors declare that the research was conducted in the absence of any commercial or financial relationships that could be construed as a potential conflict of interest.
